# Comparative Study of Understorey Birds Diversity Inhabiting Lowland Rainforest Virgin Jungle Reserve and Regenerated Forest

**DOI:** 10.1155/2013/676507

**Published:** 2013-12-26

**Authors:** Ezyan Nor Hashim, Rosli Ramli

**Affiliations:** Institute of Biological Sciences, Faculty of Science, University of Malaya, 50603 Kuala Lumpur, Malaysia

## Abstract

A comparative study of understorey birds inhabiting different habitats, that is, virgin jungle reserve (VJR) and regenerated forest (RF), was conducted in Ulu Gombak Forest Reserve and Selangor and Triang Forest Reserve, Negeri Sembilan, Peninsular Malaysia. The objective of this study was to assess the diversity of understorey birds in both habitats and the effects of forest regeneration on the understorey bird community. The mist-netting method was used to capture understorey birds inhabiting both habitats in both locations. Species composition and feeding guild indicated that understorey bird populations were similar in the two habitats. However, the number of secondary forest species such as Little spiderhunter (*Arachnothera longirostra*) in VJR is increasing due to its proximity to RF. This study discovered that RFs in both study areas are not yet fully recovered. However, based on the range of species discovered, the RFs have conservation value and should be maintained because they harbour important forest species such as babblers and flycatchers. The assessment of the community structure of understorey birds in VJR and RF is important for forest management and conservation, especially where both habitats are intact.

## 1. Introduction

Tropical forest harbours the most diverse range of flora and fauna in the world [1–3]. Heavy utilization of the natural resources of tropical forests for economic purposes (timber exploitation and plantation) has become a major threat to the tropical ecosystem [1]. Deforestation is the main issue in developing countries (such as Malaysia) as they seek to meet the basic needs of an increasing human population [2, 4]. Approximately 5 million hectares of forest cover in Malaysia have been lost between 1983 and 2003 [5]. Habitat loss has seriously threatened the survival of tropical forest birds which depend on a healthy forest ecosystem [3]. Many studies of the effects of logging on tropical forest birds inhabiting lowland rainforest have been conducted in tropical areas [1, 3, 6–13].

The lowland rainforests of Malaysia are home to 311 species of birds and the forest harbours more bird species than degraded habitat [14]. Habitat changes have influenced the community structure of understorey birds [1, 11, 15]. Several primary forest birds such as babblers and bulbuls can be used as indicator groups to evaluate the degree of habitat disturbance [3, 8, 10, 11, 16, 17]. Previous studies have indicated that species composition of the understorey bird community in the early stages of forest regeneration (less than 10 years after disturbance) is dominated by colonizer species such as spiderhunters and bulbuls [1, 3]. The recovery rate of forest specialist birds in regenerated forest (RF) increases with time when the regenerated forests are left untouched for about 20–40 years [6].

Primary forest is a fragile ecosystem that contains a higher density of rare species than RF [12]. The undisturbed habitat functions as a refuge which helps forest-dependent birds survive in a harsh environment, especially when surrounding forest is logged. VJRs have existed in Peninsular Malaysia since 1947 and there are currently 120 VJRs covering an area of 111,880 hectares [18]. These forests have been preserved strictly for research purposes including biodiversity and genetic conservation. The function of primary forest may also be supported by surrounding secondary forest if that forest is allowed to regenerate [19].

Most studies of tropical birds have focused on the early stages of forest regeneration [7, 8]. However, no study has compared the structure of bird populations in undisturbed forest remnants (which have been preserved as virgin jungle reserve) and RF (with more than 20 years of logging history). The aim of this study was to investigate the community structure of understorey birds inhabiting two RFs (logged 6–50 years ago) and two lowland rainforest VJRs in Peninsular Malaysia. The study aimed to determine the effect of habitat changes and forest regeneration on the understorey bird community structure of the two different habitats in two different forest reserves. The study examined the role of VJRs in maintaining forest bird populations, especially when they are surrounded by disturbed forest. Assessment of understorey birds inhabiting RF was used to investigate whether this habitat has the ability to conserve forest-dependent species, especially when it is located near VJR. It is important to monitor forest birds inhabiting forest remnants in order to manage forests more efficiently as well as for conservation purposes [20].

## 2. Materials and Methods

### 2.1. Study Areas

**Figure 1:**
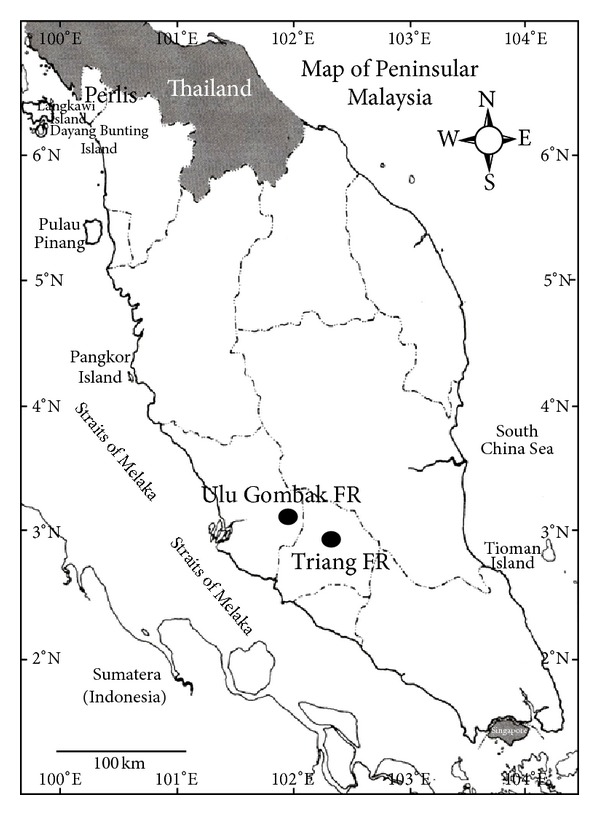
Map showing location of the two study sites in Peninsular Malaysia and Ulu Gombak Forest Reserve (UGFR), Selangor, and Triang Forest Reserve (TFR), Negeri Sembilan.

Understorey birds inhabiting two forest reserves in Peninsular Malaysia (Figure 1) were sampled from January 2009 to December 2010. Both forest reserves are lowland dipterocarp forest that has two different habitats, that is, VJRs and RFs, with a history of at least 6 years of logging.

### 2.2. Ulu Gombak Forest Reserve, Selangor

The Ulu Gombak Forest Reserve (UGFR) is located 30 kilometres from Kuala Lumpur on the north side of the Kuala Lumpur-Bentong road (N 03°19.111′; E 101°45.912′). The topography of the area is rough, with steep hillside and narrow valley bottoms. Altitude ranges from 100 to 800 m. The mean annual temperature recorded was 26-27°C. The relative humidity (83–85%) was high and mean annual rainfall is 223–227 mm [9]. The size of the forest reserve is approximately 17,000 hectares. Of this, 499 hectares are VJR. The remaining forest was logged 50 years ago before it was designated as forest reserve. The area surrounding the forest reserve is highly developed because it is located adjacent to the Karak Highway, one of the country's major highways, leading to the eastern part of Peninsular Malaysia. The forest was listed as one of the country's most popular bird watching spots [21] and important bird areas [22].

### 2.3. Triang Forest Reserve, Negeri Sembilan

Triang Forest Reserve (TFR) is located about seven kilometres from Kuala Klawang town in Jelebu, Negeri Sembilan (N 02°55.631′; E 102°07.814′). This forest is a lowland area with an altitude range of 90–500 m. The mean annual temperature is 26-27°C, with mean relative humidity of 79% and mean annual rainfall of 123–130 mm [9]. The size of the forest reserve is about 50,000 hectares. The forest was designated as productive forest reserve in 1909. The actual areas of VJR and logged forest are not available. The unlogged forest is separated from RF by a river and logging track. The most recent logging activity in the sampling area was recorded in 2005, but logging activity is still occurring in some part or parts of the forest. The forest reserve is surrounded by rubber (*Hevea brasiliensis*) plantations and human settlement. Indigenous people utilize the forest resources such as rattan (Calameae) and bamboos (Bambuseae). The forest is also used by the Malaysian army as a training area.

### 2.4. Bird Sampling

Twenty mist nets measuring 12 m × 2.5 m with mesh size of 36 mm^2^ were set up randomly within RF and VJR. Nets were set up 0.5 m above ground level and about 30 m apart. The mist-netting method is suitable for capturing understorey forest birds which are usually inconspicuous and rarely give distinctive calls [17]. Mist netting was carried out for a total of 144 days on both study sites: 72 days in VJR and 72 days in RF. All nets were operated from 0700 hours to 1800 hours. The nets were checked hourly to extract captured birds. All nets were opened for 3 consecutive days during each visit. All captured birds were identified by species, ringed with an aluminium ring bearing a serial number, morphologically measured and weighed. Captured birds were released close to the point of capture to reduce the disturbance to their daily activity.

### 2.5. Feeding Guild

During species identification, each bird was assigned to a feeding guild based on preferred diet. Guilds are communities of species utilizing particular resources in a similar manner [11]. Analysis of guilds provides information on how other guild members are likely to respond to changes in a particular habitat [11]. Six major feeding guilds were distinguished: carnivorous (CR), insectivorous (IN), frugivorous (FR), nectarivorous (NEC), omnivorous (OM), and granivorous (GR). Omnivorous species are generalist species that show characteristics of more than one feeding guild, for example, frugivorous/insectivorous (FR/IN), nectarivorous/insectivorous (NEC/IN), frugivorous/nectarivorous (FR/NEC), and frugivorous/nectarivorous/insectivorous (FR/NEC/IN).

### 2.6. Data Analysis

Community diversity refers to species richness, abundance or some combination of these in a community [23]. To compare total number of individuals and species recorded from the study sites, various indices such as the Berger-Parker Index of dominance, Shannon-Weiner Index (*H*′), and Simpson's Evenness Index (*E*) were calculated using Species Diversity and Richness Program Version 4. A species accumulation curve was plotted for both habitats in both study sites to illustrate the completeness of sampling efficiency. The first-order Jackknife method was used to estimate projected species richness at increasing levels of sampling effort. The Sorensen Similarity Coefficient Index was used to measure the similarity of understorey bird species composition in the two habitats or two study sites. The index focuses on the species common to both sites rather than those found at only one particular site.

### 2.7. Statistical Analysis

Significant differences between the group means for both habitats were determined using the Mann-Whitney *U* test. The test was chosen because sample sizes were small and samples may not have been normally distributed. The Chi-squared test was used to investigate associations between categorical variables; it is appropriate for nonparametric data which do not meet the requirements of a parametric method.

## 3. Results

### 3.1. Overall Species Richness

A total of 2,370 birds belonging to 120 species and 30 families were captured across both habitats. RF recorded higher species abundance (1,339 birds belonging to 104 species and 28 families) than VJR (1,031 birds belonging to 87 species and 24 families). According to the Shannon-Weiner Index, VJRs at both study sites are a more diverse habitat than the RFs (Table 1). Evenness indices showed that species distribution in VJR was more even than in RF. Distribution of understorey birds in the VJR of UGFR was more even than for TFR; however, distribution in RF was similar for the two study sites (Table 1).

**Figure 2:**
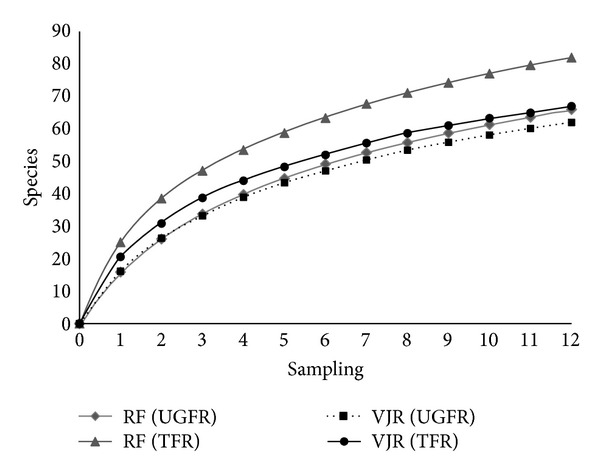
Species accumulation curve of understorey birds captured in different habitats and at different sites.

Species accumulation curves showed that bird populations in both habitats have not yet achieved asymptote (Figure 2). Extrapolated estimates of species richness indicated that 10% and 40% of forest birds remained undetected in the RF of UGFR and TFR, respectively. Similar estimates suggested that 3% and 40% of forest birds remained undetected in the VJRs of UGFR and TFR, respectively (Table 1). In both study sites diversity of understorey birds was higher in the VJRs than in the RFs, but the abundance of species was not significantly different in the two habitats (Mann-Whitney *U* test, df = 1, *P* = 0.263).

### 3.2. Species Composition

The Pycnonotidae (15 species), Timaliidae (16 species), and Muscicapidae (17 species) families made up the biggest proportion of species captured in both habitats. The number of species from these families was slightly lower in VJR (10, 16, and 13, resp.). About 62% of total abundance of understorey birds in both habitats was contributed by members of the Nectariniidae (729 birds), Timaliidae (382 birds), and Pycnonotidae (363 birds) families. The most abundant species were Little spiderhunter (*Arachnothera longirostra*), Grey-throated babbler (*Stachyris nigriceps*), and Grey-cheeked bulbul (*Alophoixus bres*). These species were among 71 species recorded in both habitats.

The abundance of Little spiderhunter was significantly higher in RF (*χ* ^2^ = 3.89, df = 1, *P* < 0.05) while the abundance of Grey-throated babbler and Grey-cheeked bulbul was significantly higher in VJR (*χ* ^2^ = 5.59, df = 1, *P* < 0.05 and *χ* ^2^ = 5.81, df = 1, *P* < 0.05, resp.) (Table 2). The abundance of near-threatened species such as Buff-necked woodpecker (*Meiglyptes tukki*) and Chestnut-naped Forktail (*Enicurus ruficapillus*) was significantly higher in RF than in VJR (*χ* ^2^ = 5.59, df = 1, *P* < 0.05 and *χ* ^2^ = 5.67, df = 1, *P* < 0.05, resp.). The abundance of vulnerable species, Brown-chested jungle flycatcher (*Rhinomyias brunneatus*), was significantly higher in VJR (*χ* ^2^ = 4.36, df = 1, *P* < 0.05) (Table 2). Twenty-five of the species recorded are classified as near-threatened or vulnerable on the IUCN Red List classification. These species showed a wide range of representation in our samples, ranging from a single individual to 48 birds. Seven species of understorey birds that are classified as near-threatened were captured only in RF and 4 species were recorded only in VJR.

### 3.3. Forest Birds in RFs

Of 104 species recorded in RF, 44 species (1,191 birds) were present in both forest reserves (Sorensen similarity coefficient = 60%). Most of these common species were recorded in TFR (Mann-Whitney *U* test, df = 1, *P* = 0.01). Little spiderhunter which was abundantly captured in TFR (*χ* ^2^ = 3.15, df = 1, *P* < 0.05) was the dominant common species. RF of TFR was dominated by species such as Rufous-backed kingfisher (*Ceyx rufidorsa*) (*χ* ^2^ = 3.15, df = 1, *P* < 0.05) and Red-eyed bulbul (*Pycnonotus brunneus*) (*χ* ^2^ = 1.03, df = 1, *P* < 0.05). Near-threatened species such as Chestnut-naped Forktail were commonly found in RF of UGFR (*χ* ^2^ = 10.23, df = 1, *P* < 0.05). UGFR harboured significant numbers of Grey-throated babbler (*χ* ^2^ = 29.32, df = 1, *P* < 0.05) and Grey-headed canary flycatcher (*Culicicapa ceylonensis*) (*χ* ^2^ = 11.03, df = 1, *P* < 0.00). Some species, for example, Yellow-bellied warbler (*Abroscopus superciliaris*), were only recorded in UGFR while Asian paradise flycatcher (*Terpsiphone paradisi*), Brown-chested jungle-flycatcher, and Sooty-capped babbler (*Malacopteron affine*) were only recorded in TFR.

### 3.4. Forest Birds in VJRs

A total of 41 species (888 individuals) were recorded in VJR across both study sites (Sorensen similarity coefficient: 46%). The abundance of similar species was not significantly different for the two study sites (Mann-Whitney *U* test, df = 1, *P* = 0.25). Little spiderhunter was the most abundant species recorded (90% of all captured birds) in TFR (*χ* ^2^ = 23.54, df = 1, *P* < 0.05). The abundance of Yellow-bellied bulbul (*Alophoixus phaeocephalus*) and Scaly-crowned babbler (*Malacopteron cinereum*) was significantly higher in TFR (*χ* ^2^ = 1.72, df = 1, *P* < 0.05 and *χ* ^2^ = 4.6, df = 1, *P* < 0.05 resp.). Greater numbers of Rufous-backed kingfisher, (*χ* ^2^ = 3.93, df = 1, *P* < 0.05), Grey-throated babbler (*χ* ^2^ = 20.99, df = 1, *P* < 0.05), and Hairy-backed bulbul (*Tricholestes criniger*) (*χ* ^2^ = 7.34, df = 1, *P* = 0.05) were captured in UGFR. Some species have limited distribution. These include Yellow-bellied bulbul which was captured only in UGFR while Asian paradise flycatcher and Chestnut-winged babbler (*Stachyris erythroptera*) were only recorded in TFR.

### 3.5. Feeding Guild Composition

IN birds are the feeding guild most frequently captured in both habitats, in terms of both individuals and species with 793 birds (33%) of 67 species. RF has a slightly greater proportion of insectivorous birds (18%) than VJR (15%) (Table 3). However, NEC/IN birds were more frequent in RF than in VJR with 454 individuals from 4 species. The abundance of FR/IN, FR/NEC, and FR/NEC/IN birds was also greater in RF while CR and FR birds were more frequent in VJR (Table 3).

## 4. Discussion

Avian community structure is closely related to variation in habitat structure, food abundance, microclimatic changes, and spatial variation [1, 11, 16, 24, 25]. Some species that inhabit unlogged forest—VJR—may persist or reappear in RF although their abundance may not be the same as in unlogged forest [8]. Regenerated forests are dominated by species that are tolerant of a wide range of habitat conditions [6]. In this study, RFs have a less diverse community of understorey birds than VJR due to the dominance of secondary forest species such as Little spiderhunter. This species is a common visitor to bananas (Musaceae) and gingers (Zingiberaceae) which grow abundantly in logged forest [8, 26].

Recolonization of understorey birds in RFs is dependent on microclimatic conditions in the understorey [8]. A large number of primary forest birds such as Grey-throated babbler and Chestnut-winged babbler recorded in RFs showed that the forests are in the process of recovery. Over 50% of captured species were present in both habitats; the species common to both habitats were predominantly common understorey families such as Timaliidae, Pycnonotidae, and Muscicapidae. Although the abundance of these common species is lower in RF, it shows that this habitat could play a larger role in conserving forest birds if the forests were allowed to regenerate further. Regenerated forest which is left untouched for a minimum period can become a surrogate habitat for forest-dependent species [19]. The community structure of understorey birds in RF can be very similar to that of primary forest after a sufficient regeneration period [3, 24].

Previous studies had shown that primary bird species such as Grey-chested jungle flycatcher (*Rhinomyias umbratilis*), Grey-headed canary flycatcher and White-crowned Forktail (*Enicurus leschenaulti*) were absent from RF 12 years after disturbance [7, 8]. However, in this study these species were successfully recorded in early stage RF, that is, TRF (approximately 5 years after disturbance). This result shows that RF was slowly recovering after having been selectively logged and that some forest birds have successfully survived in this habitat. This study also recorded ground-dwelling forest species such as Hooded pitta (*Pitta sordida*) in early stage RF (TFR) and Eyebrowed wren-babbler (*Napothera epilepidoata*) in old-growth RF (UGFR). Previous studies have found that these species were absent from RF 12 years after disturbance [8, 11]. The presence of primary forest species, even in small numbers, indicate, that the RF has the ability to support forest-dependent species. These species, which use the understorey for nesting, are very sensitive to habitat disturbance and are therefore at higher risk of extinction [11].

Different species require different periods to adapt and establish a population in RF. Some primary forest species may exploit adjacent habitat (secondary forest or degraded habitat) as a food resource whilst remaining dependent on primary forest for nesting and breeding [10, 27]. Studies of degraded habitats in Java have shown that less than 50% of persisting lowland species breed in moderately degraded habitat [12]. Regenerated forest can play an important role in conserving tropical forest birds if the forest is allowed to regenerate for sufficient time [6].

The remaining untouched section of forest reserve (known as VJR) has greater capacity to support forest birds. The presence of primary forest species such as Brown-chested jungle flycatcher, Grey-throated babbler, and Chestnut-winged babbler indicates species persistence in undisturbed habitat. A good quality VJR (of sufficient size to provide adequate food resources) or other suitable habitat can provide a breeding ground for forest-dependant species such as Green broadbill (*Calyptomena viridis*). This bird was recorded nesting in VJR of TFR during sampling. However, numerous secondary forest species such as Little spiderhunter and Red-eyed bulbul were also recorded in VJR of TFR. This is because the forest is in the early stages of regeneration. In addition, the close proximity of VJR and RF (approximately one kilometre) at this site may allow forest edge species to exploit resources available in adjacent forest habitat.

Food abundance is an important factor in avian phenology and can be affected by logging activities [24]. Breeding cycles and moulting in highly sensitive understorey birds such as insectivorous species are dependent on the availability of food resources (arthropods, fruits, and flowers). In terms of feeding guild composition, the abundance of insectivorous birds was similar in VJR and RF. In logged forest extensive growth of succession species such as banana (Musaceae) and gingers (Zingiberaceae) helps to create the cool and humid conditions that characterise VJR understorey [8]. These conditions not only attract generalist species (NEC/IN) but also allow reestablishment of understorey species such as foliage-gleaning insectivores (such as *Stachyris* babblers) [8]. This study successfully captured high numbers of bark-associated species (such as Buff-necked woodpecker) in RF. A previous study showed that woodpeckers abundance is low even after 12 years of forest regeneration [8], indicating that this species recovers only slowly during the regeneration process.

The composition of the understorey bird communities in the two study sites indicated that logged forests were not fully recovered even 50 years after logging. This finding is in accordance with other studies that show smaller populations of understorey birds in RF 25 years after disturbance than in primary forest [8, 11]. Populations of understorey birds which are similar to those of primary forest were achieved only after 20–40 years [28] or even as many as 70 years of forest regeneration [8]. Logging cycles of less than 35 years in Peninsular Malaysia had delayed the recovery process [8, 11]. Human disturbances and development around the forest reserve may have contributed to the unsuccessful recovery process of the forest. Conservation efforts should focus on primary forest birds because there is no substitute for primary forest; even a relatively small disturbance (such as selective logging) negatively affects habitat value. Nonetheless, secondary forest should not be neglected because it can also play an important role in conservation of biodiversity, especially when it is located close to primary forest. Preservation and conservation of existing lowland forest patches are crucial to the long-term survival of avifauna in Malaysia and throughout Southeast Asia.

## Figures and Tables

**Table 1:** Diversity and species richness of understorey birds inhabiting regenerated forest (RF) and virgin jungle reserve (VJR) at two study sites, Ulu Gombak Forest Reserve (UGFR) and Triang Forest Reserve (TFR).

Habitat/study sites	*S*	*N*	*D*	*H*′	*E*	First-order Jackknife	SD	Estimated species richness (%)
RF	104	1,339	0.33	3.29	0.08	104	0	100
UGFR	66	335	0.07	3.72	0.06	74	0	89
TFR	82	1,004	0.1	2.91	0.07	134	8	61
VJR	87	1,031	0.18	3.62	0.22	144.5	8.5	60.2
UGFR	62	397	0.1	3.59	0.42	64	0	97
TFR	66	634	2.63	3.31	0.18	110	2	60

*S*: number of species; *N*: total number of individuals captured; *D*: Berger-Parker dominance; *H*′: Shannon-Weiner Index; *E*: Simpson's Evenness Index; SD: standard deviation.

**Table 2:** List of common understorey bird species which show different abundance in RF and VJR across both study sites using Chi-squared test (*χ* ^2^).

Species	RF	VJR	*χ* ^2^	*P*-value
*Terpsiphone paradisi* Asian paradise flycatcher	4	10	5.01	0.037
*Rhinomyias brunneata* Brown-chested jungle flycatcher	6	14	4.36	0.018
*Meiglyptes tukki* Buff-necked woodpecker	22	6	5.59	0.017
*Enicurus ruficapillus* Chestnut-naped forktail	18	30	5.67	0.009
*Stachyris erythroptera* Chestnut-winged babbler	35	13	6.85	0.020
*Chalcophaps indica* Emerald Dove	14	24	5.42	0.016
*Alophoixus bres* Grey-cheeked bulbul	34	45	5.81	0.018
*Stachyris nigriceps* Grey-throated babbler	39	46	5.59	0.048
*Arachnothera longirostra* Little spiderhunter	436	187	3.89	0.000
*Anthreptes simplex* Plain Sunbird	9	1	47.81	0.031
*Pycnonotus brunneus* Red-eyed bulbul	21	2	4.63	0.001
*Ceyx rufidorsa*Rufous-backed kingfisher	51	76	11.49	0.000
*Malacopteron affine* Sooty-capped babbler	4	14	13.35	0.004
*Abroscopus superciliaris* Yellow-bellied warbler	2	10	8.46	0.006

**Table 3:** Feeding guild distribution of understorey bird communities in (*P* < 0.05) RF and VJR.

Feeding guilds	RF	VJR	*χ* ^2^
Carnivorous (CR)	91	113	11.735
Insectivorous (IN)	424	369	2.963
Frugivorous (FR)	64	80	8.512
Frugivorous/insectivorous (FR/IN)	219	198	2.687
Nectarivorous/insectivorous (NEC/IN)	454	197	46.439
Frugivorous/nectarivorous (FR/NEC)	42	37	0.357
Frugivorous/nectarivorous/insectivorous (FR/NEC/IN)	44	35	0.021
